# Laterally spreading tumour of the distal stomach: a case report

**DOI:** 10.1186/s12885-018-4425-3

**Published:** 2018-05-02

**Authors:** Samiullah Khan, Lan-ping Zhu, Yujie Zhang, Xin Chen, Bang-mao Wang

**Affiliations:** 1 Department of Gastroenterology and Hepatology, Tianjin Medical University General Hospital, 154 Anshan Road Heping District, 300052 Tianjin, People’s Republic of China; 2 Department of Pathology, Tianjin Medical University General Hospital, Tianjin, People’s Republic of China

**Keywords:** Laterally spreading tumour, High-grade intraepithelial neoplasia, Early gastric cancer

## Abstract

**Background:**

Laterally spreading tumours (LSTs) are superficial neoplasms that usually extend laterally along the intra-luminal wall of the gastrointestinal tract. Recently, the incidence of LSTs in the colorectal mucosa has greatly increased. However, LSTs in the stomach are exceedingly rare and have never been previously reported.

**Case presentation:**

Here, we report a 69-year-old male with epigastric pain and a gastric LST 6 cm in diameter located in the distal stomach and grossly extended into the duodenal bulb. The stomach lesion was initially diagnosed as high-grade intraepithelial neoplasia, while the duodenal lesion was diagnosed as a tubulovillous adenoma. A therapeutic strategy of endoscopic submucosal dissection and distal gastrectomy was applied. The surgeries and postoperative course were uneventful, and the patient remained asymptomatic 1 year after surgery.

**Conclusions:**

This is a clinically significant case, as it provides detailed information regarding laterally spreading early gastric cancer and emphasizes the diagnostic and therapeutic approaches for early gastric cancerous lesions.

## Background

Early gastric cancer (EGC) is a well-defined gastric malignancy that is restricted to the mucosa or submucosa regardless of the presence of lymph -node metastasis [1]. EGC tumours are usually oval or round in shape [7], but the shape of laterally-spreading EGC tumours is undefined, and in our case, the EGC in the distal stomach was diagnosed as a large circumferential-type laterally extending tumour. LSTs frequently occur in the colonic mucosa and are usually defined as neoplasms > 10 mm that extend laterally along the intra-luminal wall of the GI tract [2, 3]. However, because of their extreme rarity, no explicit definition of gastric LSTs is available in the medical database. Based on a morphological endoscopy, LSTs are usually categorized into 1 of 3 groups, as follows: (1) granular-homogenous, (2) granular-nodular mixed and (3) non-granular [2, 3]. Upper GI endoscopy, computed tomography (CT), and endoscopic ultrasonography are helpful in the diagnosis of EGC.

## Case presentation

A 69-year old male with epigastric pain visited the digestive department of Tianjin Medical University General Hospital for evaluation of his symptoms. On admission, he appeared anaemic, but no change in weight loss was observed; moreover, an abdominal examination revealed mild-abdominal tenderness. All other physical examinations were essentially normal. His medical history included diabetes (15 -years) and controlled hypertension (4 -years) but did not include drug treatments, medication, alcohol, or family risk factors for any illness. The patient denied other symptoms such as regurgitation, heartburn, nausea and vomiting, anorexia, bloating, diarrhoea, constipation, and fever. Initial laboratory investigations were insignificant except a haemoglobin level of 11 g/dL and a high C-reactive protein level of 7.44 mg/dL.

**Fig. 1:**
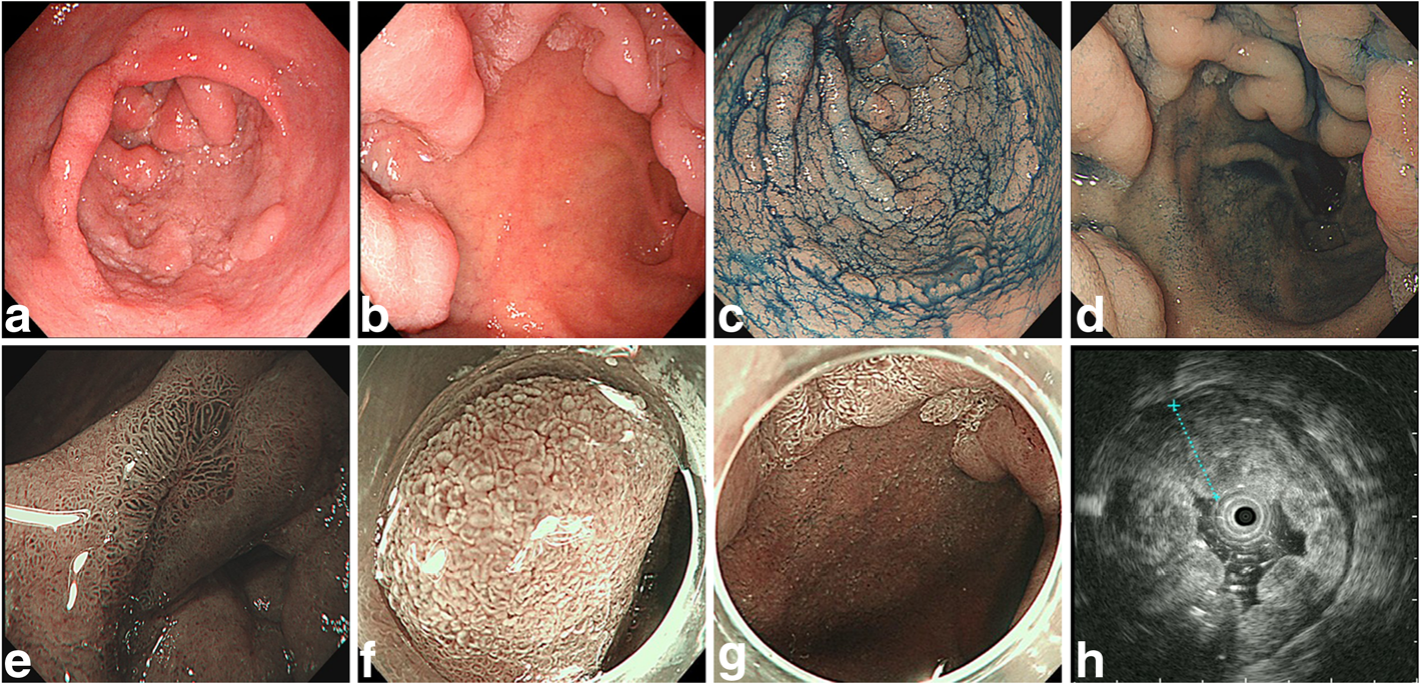
White light endoscopy showed a gastric laterally spreading tumour of the distal stomach of the granular nodular-mixed type, approximately 6 –cm in size, located at the antrum and pyloric region of the stomach; a 0-IIa + 0-IIC and 0-I lesion (**a**), along with a polypoid duodenal bulb lesion (**b**). Chromoendoscopy using indigo showed clear margins of the distal stomach (**c**) and duodenal bulb lesion (**d**). Narrow-band imaging revealed an irregular pit pattern of IIIL to IV and increased vascularization in the surrounding mucosa (**e**). A white opaque substance with oedematous villi is clearly seen in the antrum near the pyloric region (**f**), and duodenal bulb (**g**) indicated early gastric cancer. Endoscopic ultrasound showed a thickening of the mucosal layer of approximately 12 mm (**h**)

An upper GI endoscopy was performed, which showed a superficially elevated granular-nodular mixed and circumferential-type flat elevation in the distal stomach. The endoscopy also showed primary involvement of the antrum and pyloric region as well as a type 0-IIa + 0-IIc and a 0-I tumour (according to Paris classification) 6 –cm in diameter that extended into the duodenal bulb (Fig. 1a, b, c, d). Inspection with narrow band imaging revealed microvascular and microsurface changes and an irregular pit pattern of IIIL to IV; a white opaque substance was prominent in the antrum region of the stomach and duodenal bulb **(**Fig. 1e, f, g). Endoscopic ultrasound showed thickened hypoechoic mucosal, muscular mucosal and submucosal layer. The muscularis propria was intact (Fig. 1h). Enhanced abdominal CT revealed thickened gastric antral wall with prominent small lymph-nodes, a hepatic cyst, and a stone and cyst in the right kidney. Neither distant metastasis nor lymph -node invasion was observed. Abdominal ultrasound showed fatty liver and the wall of the gall bladder was not smooth.

**Fig. 2:**
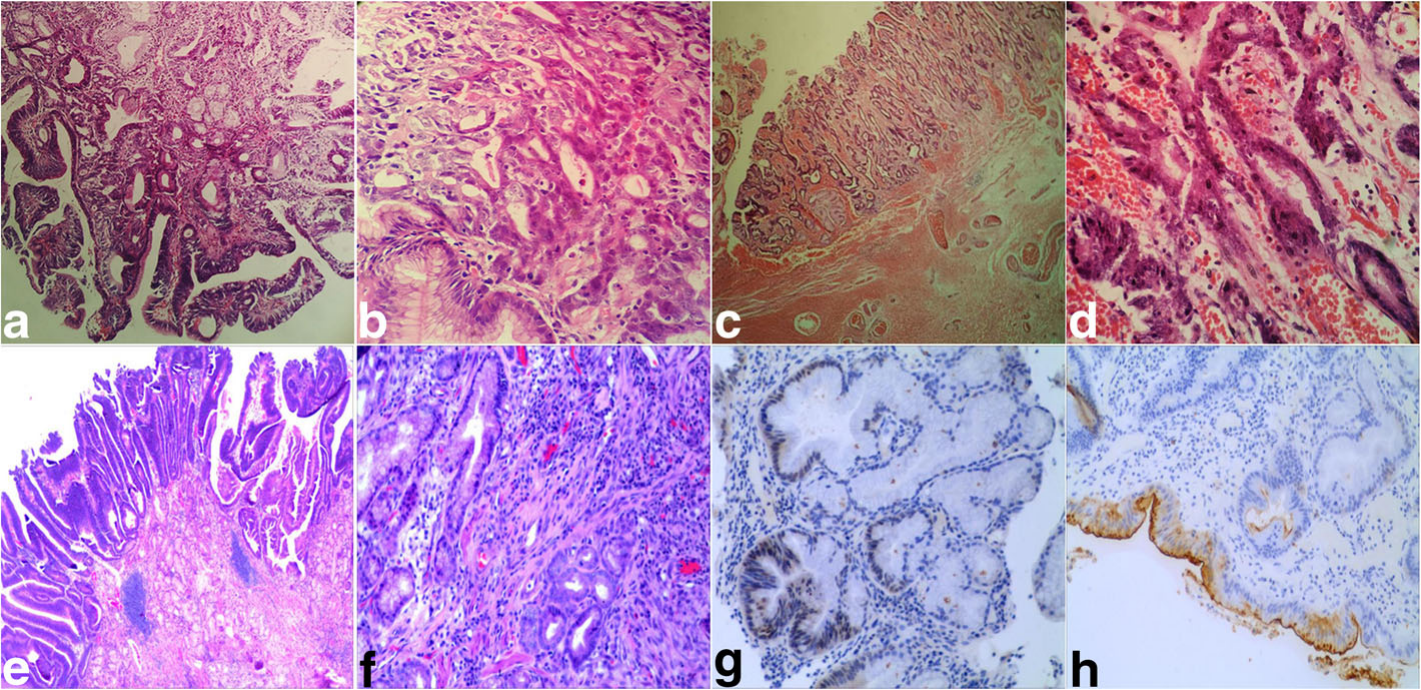
Histopathological examination of the early biopsy revealed high-grade intraepithelial neoplasia of the gastric antrum near the greater curvature and tubulovillous adenomatoid hyperplasia with low-grade intraepithelial neoplasia in the antrum and pyloric region. The histopathologic assessment showed tubulovillous hyperplasia with moderate atypical hyperplasia of the glands with crowded cells in the antropyloric region at 100× (**a**). High magnification 400× showed disruption of polarity, extremely disordered glandular structures, deep staining that, infiltrated the stroma, crowded cells, and hyperchromatic nuclei (**b**). Histopathology of the ESD showed a disordered glandular structure and polarity, visible branching growth; damaged muscular mucosal layer, and expanded submucosal blood vessels at 40× (**c**). The nuclei are deeply stained and crowded at 400× (**d**). The surgical specimen is representative of showing highly to -moderately differentiated adenocarcinoma of the gastric antrum involving the muscularis mucosa, and of a tubulovillous adenoma of the pylorus. The glands are irregular with villous growth, visible branching and structural changes (**e**). The interstitial connective tissue is visible, and inflammatory cells are leaching (**f**). Immunostains showed that a portion of the glands are CEA positive (**g**), and CDX-2 positive (**h**)

Multiple targeted biopsies of the lesion revealed chronic mucosal inflammation, erosion, high-grade intraepithelial neoplasia of the gastric antrum near the greater curvature, tubulovillous adenoma of low-grade intraepithelial neoplasia in the pyloric region and tubulovillous adenoma with glandular hyperplasia in the duodenal bulb (Fig. 2a, b). The patient was negative for *H. pylori*. Based on the endoscopic examination, pathological results, and CT findings, an EGC was suspected.

### Treatment strategy

This case was debated at a multi-disciplinary meeting. Due to the suspicion of an early gastric adenocarcinoma and the large tumour size, the team agreed that surgical resection was the best treatment. The patient was advised to undergo a surgical procedure, but he refused; then, despite the potential risk and prolonged operation time, he agreed to undergo endoscopic submucosal dissection (ESD).

#### ESD treatment for the gastric-duodenal bulb lesion

The patient underwent an uncomplicated, prolonged (15 h) ESD of the distal stomach and duodenal bulb, which was performed by an expert endoscopist. The patient was then transferred to the intensive care unit. Grossly, the resected tissue measured 6.0  × 5.0 cm. The pathologic examination of the resected tissue of the gastric antrum near the pylorus revealed extensive high-grade intraepithelial neoplasia, multifocal cancer that presented as intramucosal carcinoma, and a highly to -moderately differentiated adenocarcinoma. The lateral margins of the antrum and the pyloric region were positive for low-grade intraepithelial neoplasia (Fig. 2c, d). The remaining gastric mucosa showed severe atrophic gastritis and intense intestinal metaplasia accompanied by mild atypical hyperplasia. Based on these findings, the patient was scheduled for an additional gastrectomy.

#### Radical distal gastrectomy for gastric cancer (Billroth II anastomosis)

Two weeks later, the patient underwent radical distal gastrectomy (Billroth II anastomosis) for gastric cancer and subsequent abdominal drainage. Excisions were made to the most distal part of the stomach and part of the duodenum. The pathologic assessment of the excised tissue revealed a highly to -moderately differentiated adenocarcinoma of the gastric antrum, which involved the muscular mucosa, and tubulovillous adenoma of the pylorus and duodenal bulb (Fig. 2e, f). The duodenal bulb was non-neoplastic. An immunohistochemical study revealed that the adenoma was CEA positive and, CDX-2 positive (Fig. 2g, h). No cancer cell invasion was observed at the proximal end or at the end of the duodenal bulb. The tumour depth was limited to the muscular mucosal layer, and lymphatic-vascular involvement was absent according to haematoxylin and eosin stain.

Postoperatively, the patient had an uneventful recovery, and he was discharged on the 15th postoperative day in a stable condition. Follow-up after discharge was uneventful, and he remained asymptomatic 1 year after the surgery.

## Discussion and conclusions

This report describes an extremely rare case of large laterally-spreading EGC of the distal stomach. LSTs are considered non-polypoid lesions with specified clinic-pathologic features. They are characterized by lateral progression along the intra-luminal wall, where they form broad-based granular or non-granular flat-type lesions [2]. EGCs progress slowly, although the speed of their progression is accelerated according to the downward invasion by the cancer. Most patients with EGC are usually asymptomatic or they present with the earliest signs and symptoms of indigestion, loss of appetite, heartburn, epigastric pain, and anaemia.

The aetiology of gastric cancer is multifactorial and involves both hereditary predilection and environmental aspects. Family history, genetic risk factors, *H. pylori* and Epstein–Barr virus infection [4], pernicious anaemia, chronic atrophic gastritis, Ménétrier’s disease, smoking and dietary factors, such as the consumption of processed foods and pickled vegetables, as well as obesity are risk factors for gastric cancer. Elevated expression rates of tumour markers such as CEA and CA 19–9, and recently, cancer-testis antigens have been reported in gastric cancer; which include Kita-kyushu lung cancer antigen 1, which is expressed in; 79.5%; MAGE-A1 at; 32.5%, MAGE-A3 at; 39.8%, and NY-ESO-1 at; 15.7% [5]. According to Futawatari et al. [5] Kita-kyushu lung cancer antigen 1 is tumour-specific and can be frequently detected even in the early stages of the disease. In addition, the assessment of cancer-testis antigens expression in gastric mucosa infected with *H. pylori* may be helpful for the identification of patients with a high risk of gastric cancer.

**Fig. 3:**
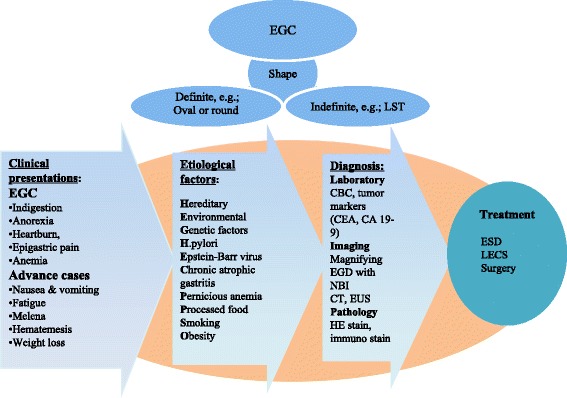
A summary of the clinical and aetiological factors and the management of early-gastric cancer lesions. (EGC; early gastric cancer, LST; laterally spreading tumour, EGD; Oesophagogastroduodenoscopy, CBC; complete blood count, NBI; narrow band imaging; CT; computed tomography, EUS; endoscopic ultrasound, HE; haemoxylin and eosin, CEA; Carcinoembryonic antigen, CA 19–9; Carbohydrate Antigen 19–9, ESD; endoscopic submucosal dissection, LECS; Laparoscopic and endoscopic cooperative surgery).

The present case is unique and suggestive. This was the first gastric LST of the distal stomach that extended into the entire duodenal bulb; this was also the second gastric LST to be reported after a laterally-spreading gastric mucosal cancer was reported by Nunobe et al. [6]. Nunobe demonstrated surgical techniques for safe lesion removal, but the case report lacked genuine images of the lesion as well as detailed information on the lesion. In contrast, this case presents serial endoscopic and histopathological images that demonstrate not only the gross-morphological changes of the lesion but also a thorough examination of the pit patterns and surface patterns of the lesion, respectively. In this case, the circumferential-type laterally spreading tumour in the antrum adjacent to the pyloric mucosa exhibited roughness and granulation, and macroscopically, the nodules were confined to the pylorus and duodenal bulb. In addition, oedematous villi with a white opaque substance were prominent in the antral and duodenal bulb regions of the lesion which indicates EGC. According to Um et al. [7] clinical behaviors of EGC vary by the endoscopic gross appearance. Endoscopic gross appearance associated with histological differentiation and clinical behavior of EGC. In order to characterize tumour burden of EGC, usually the longest diameter of tumour and maximal longitudinal diameter of tumour, the tumour burden is important to predict clinical behaviors of cancer. In contrast, the clinicopathologic characteristics of LST are also dependent on the endoscopic appearance of the lesion surface, that is, LST-granular or granular nodular mixed as compared with LST-non granular, and the risk of malignant transformation is significantly higher for the mixed nodular or pseudo-depressed subtypes and lesion of larger size [8]. According to Ohara et al. [1] endoscopic treatment for superficial elevated and depressed type EGC has a high potential to result in non-curative resection. Based on the classification of colorectal LSTs, LST- non-granular and LST- granular-nodular mixed with larger size exhibit a high risk of submucosal invasion and malignancy [2, 3]. Thus, these subtypes of tumours are suggestive to undergo surgery rather than incomplete endoscopic resection [8]. A summary of the clinical and aetiological factors as well as the management of EGC lesions is presented in (Fig. 3) [1, 4, 5].

Detection of early neoplasia, precise characterization of the lesion, and decision-making as to the ultimate treatment are crucial for better outcomes [9]. Therefore, EGC has driven the development of novel imaging technologies such as NBI and high-definition chromoendoscopy using indigo to detect early lesions [9]. A methodical approach to describe and report superficial lesions so that the appropriate resection approach is performed includes the location of the lesion, its size, morphology, surface pattern, as well as other gross morphological features [2].

Considering that ESD is broadly recognized as a less invasive method for the treatment of EGC [9], it is still a complicated procedure and is accompanied by a negligible risk of lymph node metastasis, bleeding, perforation, and longer operation times [6]. Moreover, ESD may result in incomplete or non-curative resection in EGC patients, which may necessitate additional gastrectomy [1, 10]. According to Ohara et al. [1] ESD may be curative in (a) differentiated-type mucosal cancers that are negative for ulceration (UL (−)) and without lymphovascular invasion irrespective of tumour size; (b) differentiated-type UL(+) mucosal cancers without lymphovascular invasion and a size ≤ 30 mm; (c) undifferentiated-type UL(−) mucosal cancers without lymphovascular invasion and a size ≤ 20 mm; (d) differentiated-type submucosal cancers ≤ 500 μm without lymphovascular invasion and those ≤ 30 mm. ESD is non-curative for undifferentiated-type lesions > 20 mm and superficial elevated and depressed-type 0-IIa + IIc or 0-IIc + IIa, and UL(+) lesions. Thus, endoscopic resection of these tumours is considered an investigational treatment as in our case, and additional surgical treatment should be performed [10, 11].

According to the 2010 Japanese gastric cancer treatment guidelines (ver. 3), gastrectomy with lymph -node dissection is the standard treatment for EGC [11]. Recently, laparoscopic surgery, particularly laparoscopic-assisted distal gastrectomy for EGC, has gained an advantage to some extent over open surgery in terms of minimal invasiveness, decreased morbidity and better postoperative short and long-term patient outcomes with excellent 5-year overall survival [9, 12–15]. More recently, laparoscopic and endoscopic cooperative surgery for benign gastric tumours and EGC lesions has been promising in terms of minimal invasiveness, precise lesion localization, safe excision, minimal margins, restoration and reduction in hospital stay [6, 16]. We remain confident that, commercialization of the knowledge of EGC lesions may improve their primary detection rate and thus the treatment outcome.

This case is a new indication for the gastroenterologist as well as for researchers. Early identification and curative management of EGC lesions are crucial for better patient outcomes. Lesions, particularly those of a larger size or with granules and nodules, should be removed surgically rather than by endoscopic resection because these tumours are highly invasive and carry a significant risk of malignancy. Many clinicians and researchers hope that our knowledge will be improved based on new scientific findings, which will lead to the evolution of gastric cancer diagnosis and treatment.

## Abbreviations

CA 19–9: Carbohydrate Antigen 19–9
CEA: Carcinoembryonic antigen
CT: Computed tomography
EGC: Early-gastric cancer
ESD: Endoscopic submucosal dissection
LST: Laterally spreading tumours

## References

[1] Ohara Y, Toshikuni N, Matsueda K, Mouri H, Yamamoto H (2016). The superficial elevated and depressed lesion type is an independent factor associated with non-curative endoscopic submucosal dissection for early gastric cancer. Surg Endosc.

[2] Vleugels JLA, Hazewinkel Y, Dekker E (2017). Morphological classifications of gastrointestinal lesions. Best Pract Res Clin Gastroenterol.

[3] Miyamoto H, Ikematsu H, Fujii S, Osera S, Odagaki T, Oono Y, Yano T, Ochiai A, Sasaki Y, Kaneko K (2014). Clinicopathological differences of laterally spreading tumors arising in the colon and rectum. Int J Color Dis.

[4] Matsusaka K, Funata S, Fukayama M, Kaneda A (2014). DNA methylation in gastric cancer, related to helicobacter pylori and Epstein-Barr virus. World J Gastroenterol : WJG.

[5] Futawatari N, Fukuyama T, Yamamura R, Shida A, Takahashi Y, Nishi Y, Ichiki Y, Kobayashi N, Yamazaki H, Watanabe M (2017). Early gastric cancer frequently has high expression of KK-LC-1, a cancer-testis antigen. World J Gastroenterol.

[6] Nunobe S, Hiki N, Gotoda T, Murao T, Haruma K, Matsumoto H, Hirai T, Tanimura S, Sano T, Yamaguchi T (2012). Successful application of laparoscopic and endoscopic cooperative surgery (LECS) for a lateral-spreading mucosal gastric cancer. Gastric Cancer.

[7] Um YJ, Kim HW, Jung DH, Kim J-H, Park JJ, Youn YH, Park H, Kim JW, Choi SH, Noh SH (2017). The longest diameter of tumor as a parameter of endoscopic resection in early gastric cancer: in comparison with tumor area. PLoS One.

[8] Kim KO, Jang BI, Jang WJ, Lee SH (2013). Laterally spreading tumors of the colorectum: clinicopathologic features and malignant potential by macroscopic morphology. Int J Color Dis.

[9] Takahashi T, Saikawa Y, Kitagawa Y (2013). Gastric Cancer: current status of diagnosis and treatment. Cancers.

[10] Yamada T, Sugiyama H, Ochi D, Akutsu D, Suzuki H, Narasaka T, Moriwaki T, Endo S, Kaneko T, Satomi K (2014). Risk factors for submucosal and lymphovascular invasion in gastric cancer looking indicative for endoscopic submucosal dissection. Gastric Cancer.

[11] Japanese Gastric Cancer Association (2011). Japanese gastric cancer treatment guidelines 2010 (ver. 3). Gastric Cancer.

[12] Kim W, Kim HH, Han SU, Kim MC, Hyung WJ, Ryu SW, Cho GS, Kim CY, Yang HK, Park DJ (2016). Decreased morbidity of laparoscopic distal gastrectomy compared with open distal gastrectomy for stage I gastric Cancer: short-term outcomes from a multicenter randomized controlled trial (KLASS-01). Ann Surg.

[13] Katai H, Mizusawa J, Katayama H, Takagi M, Yoshikawa T, Fukagawa T, Terashima M, Misawa K, Teshima S, Koeda K (2017). Short-term surgical outcomes from a phase III study of laparoscopy-assisted versus open distal gastrectomy with nodal dissection for clinical stage IA/IB gastric cancer: Japan clinical oncology group study JCOG0912. Gastric Cancer.

[14] Villanueva MT (2014). Gastric cancer: a master KLASS in laparoscopic gastrectomy. Nat Rev Clin Oncol.

[15] Hiki N, Katai H, Mizusawa J, Nakamura K, Nakamori M, Yoshikawa T, Kojima K, Imamoto H, Ninomiya M, Kitano S (2018). Long-term outcomes of laparoscopy-assisted distal gastrectomy with suprapancreatic nodal dissection for clinical stage I gastric cancer: a multicenter phase II trial (JCOG0703). Gastric Cancer.

[16] Ntourakis D, Mavrogenis G (2015). Cooperative laparoscopic endoscopic and hybrid laparoscopic surgery for upper gastrointestinal tumors: current status. World J Gastroenterol.

